# THE RELATIONSHIP BETWEEN RESIDUAL MOOD SYMPTOMS, DISPOSITIONAL MINDFULNESS, AND QUALITY OF LIFE IN BIPOLAR DISORDER

**DOI:** 10.1192/j.eurpsy.2023.1461

**Published:** 2023-07-19

**Authors:** F. P. Piazza, B. Solé, S. Martín-Parra, A. Martínez-Arán, N. E. Fares-Otero

**Affiliations:** 1 ICN, Hospital Clinic of Barcelona; 2 ICN, IDIBAPS-CIBERSAM; 3 Facultat de Psicologia; 4 ICN, Universitat de Barcelona (UB), Barcelona, Spain

## Abstract

**Introduction:**

Bipolar disorder (BD) is a chronic and recurrent mental condition characterized by mood fluctuations between hypomania or mania and depression, with high level of burden and mortality rates (Hayes et al., 2015). Subsyndromal mood symptoms, including residual depression, mania and/or anxiety, are major risk factors for episodic relapses after mood stabilisation (Samalin et al., 2016). A psychological protective mechanism against the occurrence of these maladaptative mood symptoms is dispositional mindfulness (DM). DM refers to paying purposeful attention to present moment experiences with a curious, non-judgmental and accepting attitude (Radford et al., 2014). DM has been barely assessed in BD and there is very little evidence on the relationship between DM, residual mood symptoms and quality of life

**Objectives:**

To explore associations between DM, residual mood symptoms and quality of life in individuals with BD

**Methods:**

After informed consent, a total of 94 adults (Mean age= 45.57 years, 41.50% Male) with diagnosis of BD according to DSM-5 criteria, in full or partial remission,were recruited from the Bipolar and Depressive Disorders Unit at the Hospital Clinic of Barcelona. The ethical committee approved this study. Dispositional mindfulness was assessed using the Mindfulness Attention Awareness Scale (MAAS).The presence of residual depressive symptoms was assessed with the Hamilton Depression Rating Scale (HDRS), residual mania symptoms were assessed with the Young Mania Rating Scale(YMRS), and anxiety symptoms were assessed with the Hamilton Anxiety Rating Scale (HAM-A). The subjective quality of life was assessed with the Quality of Life in Bipolar Disorder Questionnaire (QoL-BD). Pearson correlations were carried out and the level of significance was set at p<0.05

**Results:**

DM was negatively related to residual depressive symptoms (r= -0.283; p=0.009) and to anxiety symptoms (r=-0.345; p<0.001), and positively related to quality of life (r=0.433; p<0.001), but not related to residual manic symptoms in BD

**Conclusions:**

Our preliminary data suggest that BD patients with higher levels of DM may experience less depressive and anxiety subsyndromal symptoms and perceived higher quality of life. No associations were detected regarding mania symptoms. These findings support the use of mindfulness training as an adjunct therapy to pharmacotherapy to reduce residual mood symptoms and improve quality of life in patients with BD

**Disclosure of Interest:**

None Declared

